# Marital Satisfaction, Sex, Age, Marriage Duration, Religion, Number of Children, Economic Status, Education, and Collectivistic Values: Data from 33 Countries

**DOI:** 10.3389/fpsyg.2017.01199

**Published:** 2017-07-21

**Authors:** Piotr Sorokowski, Ashley K. Randall, Agata Groyecka, Tomasz Frackowiak, Katarzyna Cantarero, Peter Hilpert, Khodabakhsh Ahmadi, Ahmad M. Alghraibeh, Richmond Aryeetey, Anna Bertoni, Karim Bettache, Marta Błażejewska, Guy Bodenmann, Tiago S. Bortolini, Carla Bosc, Marina Butovskaya, Felipe N. Castro, Hakan Cetinkaya, Diana Cunha, Daniel David, Oana A. David, Alejandra C. Domínguez Espinosa, Silvia Donato, Daria Dronova, Seda Dural, Maryanne Fisher, Aslıhan Hamamcıoğlu Akkaya, Takeshi Hamamura, Karolina Hansen, Wallisen T. Hattori, Ivana Hromatko, Evrim Gulbetekin, Raffaella Iafrate, Bawo James, Feng Jiang, Charles O. Kimamo, Fırat Koç, Anna Krasnodębska, Amos Laar, Fívia A. Lopes, Rocio Martinez, Norbert Mesko, Natalya Molodovskaya, Khadijeh Moradi Qezeli, Zahrasadat Motahari, Jean C. Natividade, Joseph Ntayi, Oluyinka Ojedokun, Mohd S. B. Omar-Fauzee, Ike E. Onyishi, Barış Özener, Anna Paluszak, Alda Portugal, Anu Realo, Ana P. Relvas, Muhammad Rizwan, Agnieszka L. Sabiniewicz, Svjetlana Salkičević, Ivan Sarmány-Schuller, Eftychia Stamkou, Stanislava Stoyanova, Denisa Šukolová, Nina Sutresna, Meri Tadinac, Andero Teras, Edna L. T. Ponciano, Ritu Tripathi, Nachiketa Tripathi, Mamta Tripathi, Maria E. Yamamoto, Gyesook Yoo, Agnieszka Sorokowska

**Affiliations:** ^1^Institute of Psychology, University of Wroclaw Wroclaw, Poland; ^2^Counseling and Counseling Psychology, Arizona State University Tempe, AZ, United States; ^3^Faculty in Sopot, SWPS University of Social Sciences and Humanities Sopot, Poland; ^4^Department of Psychiatry and Behavioral Science, University of Washington Seattle, DC, United States; ^5^Behavioral Sciences Research Center, Baqiyatallah University of Medical Sciences Tehran, Iran; ^6^Department of Psychology, King Saud University Riyadh, Saudi Arabia; ^7^School of Public Health, University of Ghana Legon, Ghana; ^8^Department of Psychology, Catholic University of Milan Milan, Italy; ^9^Department of Psychology, The Chinese University of Hong Kong Hong Kong, Hong Kong; ^10^Department of Psychology, University of Zurich Zurich, Switzerland; ^11^Graduate Program in Morphological Sciences, Federal University of Rio de Janeiro Rio de Janeiro, Brazil; ^12^Cognitive and Behavioral Neuroscience Unit, D'Or Institute for Research and Education Rio de Janeiro, Brazil; ^13^Institute of Ethnology and Anthropology, Russian Academy of Sciences Moscow, Russia; ^14^Russian State University for the Humanities Moscow, Russia; ^15^Laboratory of Evolution of Human Behavior, Federal University of Rio Grande do Norte Natal, Brazil; ^16^Department of Psychology, Faculty of Languages History and Geography, Ankara University Ankara, Turkey; ^17^Faculty of Psychology and Education Sciences, University of Coimbra Coimbra, Portugal; ^18^International Institute for the Advanced Studies of Psychotherapy and Applied Mental Health, Babes-Bolyai University Cluj-Napoca, Romania; ^19^Department of Clinical Psychology and Psychotherapy, Babes-Bolyai University Cluj-Napoca, Romania; ^20^Department of Psychology, Universidad Iberoamericana Mexico City, Mexico; ^21^Faculty of Arts and Sciences, Izmir University of Economics Izmir, Turkey; ^22^Department of Psychology, Saint Mary's University Halifax, Canada; ^23^Department of Anthropology, Cumhuriyet University Sivas, Turkey; ^24^Faculty of Psychology, University of Warsaw Warsaw, Poland; ^25^Faculty of Medicine, Federal University of Uberlândia Uberlândia, Brazil; ^26^Department of Psychology, University of Zagreb Zagreb, Croatia; ^27^Department of Psychology, Akdeniz University Antalya, Turkey; ^28^Department of Clinical Services, Federal Neuro-Psychiatric Hospital Benin-City, Nigeria; ^29^Central University of Finance and Economics Beijing, China; ^30^Department of Psychology, University of Nairobi Nairobi, Kenya; ^31^Institute of Pedagogical Sciences, University of Opole Opole, Poland; ^32^Department of Social Psychology, University of Granada Granada, Spain; ^33^Institute of Psychology, University of Pécs Pécs, Hungary; ^34^Department of Agricultural Extension and Education, Razi University Kermanshah, Iran; ^35^Institute of Psychology, University of Science and Culture Tehran, Iran; ^36^Institute of Psychology, Pontifical Catholic University of Rio de Janeiro Rio de Janeiro, Brazil; ^37^Faculty of Computing and Management Science, Makerere University Business School Kampala, Uganda; ^38^Faculty of Social and Management Sciences, Adekunle Ajasin University Akungba-Akoko, Nigeria; ^39^School of Education and Modern Languages, Universiti Utara Malaysia Sintok, Malaysia; ^40^Department of Psychology, University of Nigeria Nsukka, Nigeria; ^41^Department of Anthropology, Istanbul University Istanbul, Turkey; ^42^Department of Psychology, University of Warwick Coventry, United Kingdom; ^43^Department of Psychology, University of Tartu Tartu, Estonia; ^44^University of Karachi, Institute of Clinical Psychology Karachi, Pakistan; ^45^Center of Social and Psychological Sciences, Institute of Experimental Psychology SAS Bratislava, Slovakia; ^46^Department of Social Psychology, University of Amsterdam Amsterdam, Netherlands; ^47^Department of Psychology, South-West University “Neofit Rilski” Blagoevgrad, Bulgaria; ^48^Educational Research Center, Matej Bel University Banská Bystrica, Slovakia; ^49^Coaching Department, Universitas Pendidikan Indonesia Bandung, Indonesia; ^50^Mõttemaru OÜ Tartu, Estonia; ^51^Institute of Psychology, University of the State of Rio de Janeiro Rio de Janeiro, Brazil; ^52^Indian Institute of Management Bangalore, Organizational Behaviour and Human Resource Management Bangalore, India; ^53^Indian Institute of Technology Guwahati Guwahati, India; ^54^Department of Child and Family Studies, Kyung Hee University Seoul, South Korea; ^55^Department of Psychotherapy and Psychosomatic medicine, Technische Universität Dresden Dresden, Germany

**Keywords:** marital satisfaction, cross-cultural research, relationships, Religion and Psychology, family studies

## Introduction

Forms of committed relationships, including formal marriage arrangements between men and women, exist in almost every culture (Bell, 1997). Yet, similarly to many other psychological constructs (Henrich et al., 2010), marital satisfaction and its correlates have been investigated almost exclusively in Western countries (e.g., Bradbury et al., 2000). Meanwhile, marital relationships are heavily guided by culturally determined norms, customs, and expectations (for review see Berscheid, 1995; Fiske et al., 1998). While we acknowledge the differences existing both between- and within-cultures, we measured marital satisfaction and several factors that might potentially correlate with it based on self-report data from individuals across 33 countries. The purpose of this paper is to introduce the raw data available for anybody interested in further examining any relations between them and other country-level scores obtained elsewhere. Below, we review the central variables that are likely to be related to marital satisfaction.

### Gender

Gender has long been identified in the literature as a predictor of marital satisfaction (Bernard, 1972). Specifically, early works suggested that men report being more satisfied with their marriages compared to women in both Western (e.g., Schumm et al., 1998) and non-Western (e.g., Rostami et al., 2014) cultures. However, sex differences in marital satisfaction may differ across cultures due to traditional sex roles (Pardo et al., 2012) and larger-scale cultural variables, such as sex egalitarianism (Taniguchi and Kaufman, 2013).

### Age

Few studies have explicitly examined age effects on reports of marital satisfaction (see Schmitt et al., 2007). Thus, no clear predictions concerning age-related patterns of results can be derived from the literature. However, in some studies, age was found to be negatively related to marital satisfaction (e.g., Lee and Shehan, 1989). Importantly, age should be examined as a predictor of marital satisfaction with respect to the duration of the marriage.

### Duration of the marriage

The time that partners have spent together has been shown to correlate with marital satisfaction (Kurdek, 1999; Lavner and Bradbury, 2010). The effect of marriage length on marital satisfaction is negative (it decreases with a relationship length) or U-shaped (it decreases in the beginning and increases after some time) (Karney and Bradbury, 1995; Kurdek, 1999). One could predict that this variable may differ across cultures as, for example, in arranged marriages relationship satisfaction might be lower in the early stages of a marriage (Xiaohe and Whyte, 1990).

### Religiosity

For many cultures, religion is strongly connected to numerous relationship-related values and norms and thus it may be correlated with marital satisfaction (Call and Heaton, 1997; Fincham et al., 2011). Positive associations between religiosity and marital satisfaction have been found across different religious groups, such as Christians, Jewish, Mormons, and Muslims (Marks, 2005).

### Children

Some previous studies from various cultures revealed contradictory results regarding the correlation between the number of children and marital satisfaction (see Twenge et al., 2003; Onyishi et al., 2012). This suggests that some culture-dependent factors may influence the association between marital satisfaction and the number of children.

### Economic status

Low income or material hardship is associated with a serious threat to marital quality and stability (Lichter and Carmalt, 2009). However, some studies showed cross-cultural differences in the strength of this association (Kamo, 1993).

### Education

Few studies examine whether education level is related to marital satisfaction. For example, Janssen et al. (1998) found that highly educated women had higher rates of unstable marriages. Using the National Survey of Family Growth data, Heaton (2002) round opposite results, wherein marital dissolution was lower among women who were more educated. Therefore, the findings regarding the association between marital satisfaction and education level based primarily on Western culture are not clear and raise the question of whether such an association exists globally.

### Cultural considerations (collectivism vs. individualism)

The criteria of a satisfying marriage may vary greatly based on one's larger cultural context, specifically on whether the culture primarily identifies as a collectivistic or an individualistic one (Dillon and Beechler, 2010). Collectivistic and individualistic cultures have different cultural norms, values, and familial obligations (Hofstede, 2001). For example, fulfilling familial duties may be beneficial for marital satisfaction in a traditional Chinese marriage (Wang, 1994), whereas fulfilling hedonistic goals of husbands and wives seems to predict marital satisfaction in Western countries (e.g., Lalonde et al., 2004).

The current dataset gathers the data about marital satisfaction and its potential correlates from 33 Western and non-Western countries. We measured gender, age, duration of marriage, religiosity, number of children, economic status, education and individualism/collectivism. The dataset is introduced in order to supplement previous studies conducted typically on Westernized samples.

## Materials and methods

### Participants

Data from 7,767 individuals was collected in 33 countries: Brazil, Bulgaria, Canada, China, Croatia, Estonia, Germany, Ghana, Greece, Hong Kong, Hungary, India, Indonesia, Iran, Italy, Kazakhstan, Kenya, Malaysia, Mexico, Nigeria, Pakistan, Poland, Portugal, Romania, Russia, Saudi Arabia, Slovakia, South Korea, Spain, Switzerland, Turkey, United Kingdom, and Uganda. All participants were over the age of 18 and were currently married. Due to missing data 589 subjects were excluded. The final sample included data from 7,178 participants. On average, the participants were 40.7 years old (*SD* = 11.4), and the average marriage duration to date was 14.8 years (*SD* = 11.6).

### Procedure

The study was conducted according to the principles of the Declaration of Helsinki. The data were collected from July 2012 to December 2013 by the co-authors and their respective research teams in their home countries. All samples were convenience samples. Depending on the country, students were recruited in different ways (e.g., students, acquaintances of the researchers, participants of vocational courses, inhabitants of home towns of the researchers etc.). All participants took part in the study on a voluntary basis and provided an informed consent. The procedure across almost all study sites was identical—they completed the paper-and-pencil questionnaires with an approximate time of participation of 30 min, with an exception of two countries (Switzerland and Bulgaria) where some participants filled in the questionnaires online. In general, participants were not compensated for their participation, however participants in Hong Kong were compensated 50 Hong Kong dollars. In countries where more than one person filled in the questionnaire at the same time, we were concerned with their anonymity and the fact that they were not influencing each other. The detailed sampling strategies and research forms are presented for each country separately in Table 1.

**Table 1:** Detailed place of the study, recruitment strategy and form of the study.

**Country**	**Place of the study**	**Recruitment strategy**	**Form of the study**
Brazil	Natal, Porto Alegre, Rio de Janeiro	Students, their acquaintances and families	Group
Bulgaria	Blagoevgrad, Sofia	Students, members of the fitness club, customers in shopping malls, acquaintances of the researcher	Individual or group
Canada	Halifax	Students	Individual
China	Beijing	Students and participants recruited by HR managers of some companies	Individual or group
Croatia	Zagreb, Rijeka, Osijek, Split	Students, their acquaintance and families	Individual
Estonia	Tartu	Students, their acquaintance and families, researchers' acquaintances and neighbors	Individual
Germany	Jena, Friedrichshafen	People in a public library	Individual
Ghana	Legon	Students, their acquaintance and families, researchers' acquaintances and neighbors	Individual
Greece	Thessaloniki	People at the police station applying for issuing the passport	Individual
HongKong	Hong Kong	Students, their acquaintance and families	Individual
Hungary	Pécs	Students and people from academic community	Individual
India	Bangalore	Working executives on part-time courses	Individual or group
Indonesia	Bandung	Students, teachers, lecturers, government employees	Individual
Iran	Tehran, Kermanshah	Students, their acquaintance and families, researchers' acquaintances and neighbors	Individual
Italy	Milan	Students, their acquaintances and families, professionals at part-time courses	Individual
Kazakhstan	Kokshetau	Researcher's acquaintances and neighbors	Individual
Kenya	Nairobi	Acquaintances of the researcher and accidently met people	Individual
Malaysia	Kedah, Sintok	Students	Group
Mexico	Ciudad de Mexico	Students, their acquaintance and families, researchers' acquaintances and neighbors	Individual
Nigeria	Akungba-Akoko, Benin City, Nsukka	People at the local government offices, staff from secondary school and their acquaintances	Individual
Pakistan	Karachi	University students, faculty members and their families	Individual
Poland	Wrocław, Brzeg	Students, their acquaintance and families, researchers' acquaintances and neighbors	Individual
Portugal	Coimbra, Aveiro, Leiria, Lisboa	Students, their acquaintance and families	Individual
Romania	Cluj-Napoca	Students	Individual or group
Russia	Moscow	Students, their acquaintances and families, professionals at part-time courses	Individual
Saudi Arabia	Riyadh	Students	Individual
Slovakia	Banská Bystrica, Nitra	Students and students of the University of the Third Age	Group
South Korea	Seoul	Students, acquaintances of the researchers	Individual
Spain	Granada, Valencia	Students, their acquaintances and families, acquaintances of the researchers	Individual
Switzerland	Zurich	Students, their acquaintances and families, researchers' workplaces	Individual or group
Turkey	Ankara, Sivas, Izmir	Researchers' acquaintances and neighbors	Individual or group
U.K.	Cardiff	Researchers' acquaintances, their families, people working in services (stores, travel agencies, foodservice, banks etc.)	Individual
Uganda	Kampala	Students, their acquaintance and families, researchers' acquaintances and neighbors	Individual

The original version of the questionnaires were in English, and in all non-English speaking countries the questionnaires were translated into participants' native language by research team members fluent in English using the back-translation procedure (Brislin, 1970). Specifically, the research teams translated the measures into the native language of the participants, and then had a bilingual person back-translate the measures into English. Differences between the original English version and the back-translation were discussed, and mutual agreements were made on the most appropriate translation.

### Measures

#### Marital satisfaction

Marital satisfaction was measured with two scales to ensure that results were not dependent upon the applied questionnaire. In the first step, participants completed the Marriage and Relationships Questionnaire (MRQ) developed by Russell and Wells (1993). Specifically, the 9-item version of the MRQ (“Love Scale”) was used because it has been found to be appropriate for cross-cultural use in terms of satisfactory psychometric characteristics (Lucas et al., 2008; Weisfeld et al., 2011). Sample questions from this questionnaire included: “Do you enjoy your husband's/wife's company?”; “Do you enjoy doing things together?”; “Are you proud of your husband/wife?”. Participants answered these questions on a 5-point scale, which ranged from 1 (*yes*) to 5 (*no*). A higher number indicated higher marital satisfaction. Secondly, participants completed the Kansas Marital Satisfaction Scale (KMSS; Schumm et al., 1983; Schumm and Bugaighis, 1986), which is also a well-established tool of satisfactory psychometric characteristics (Schumm and Bugaighis, 1986; Crane et al., 2000). The KMSS has previously been validated for studies involving non-Western samples (Shek and Tsang, 1993). The scale contains 3 questions: “How satisfied are you with your marriage?”; “How satisfied are you with your wife/husband as a spouse?”; “How satisfied are you with your relationship with your wife/husband?”. Participants answered this questions on a 7-point scale, which ranged from 1 (*very dissatisfied*) to 7 (*very satisfied*). A higher number indicated higher marital satisfaction.

In order to test whether the scales were culturally equivalent, we conducted exploratory factor analysis and then compared factor score loadings obtained in each country with the pooled data using the proportionality coefficient (Tucker's Phi). We also analyzed the reliability of each scale of marital satisfaction (Table 1), and we conducted an exploratory factor analysis in each sample for the MRQ scale. One item (“*Do you love your husband/wife?”*) had low factor score loadings for several countries (Romania: −0.382; Nigeria: 0.286; Malaysia: 0.247; Kenya: 0.396), so it should be excluded from the further analysis. We then calculated the proportionality coefficient (Tucker's phi) by comparing factor score loadings of the 8-item scale between the pooled data and each sample's factor score loadings separately. The results indicated that the scale was culturally equivalent (Table 2). Cronbach's alpha for the scale calculated on the pooled data was 0.90. Results of this analysis indicated that KMSS scale was reliable and culturally equivalent (Table 2). Cronbach's alpha on the pooled data reached 0.94.

**Table 2:** Results of the analysis of cultural equivalence of the scale.

**Country**	**Cronbach's α MRQ scale**	**Tucker's Phi coefficients MRQ scale**	**Cronbach's α KMSS scale**	**Tucker's Phi coefficients KMSS scale**
Brazil	0.86	0.99	0.97	1.00
Bulgaria	0.91	1.00	0.76	1.00
Canada	0.94	1.00	0.96	1.00
China	0.88	1.00	0.93	1.00
Croatia	0.89	1.00	0.96	1.00
Estonia	0.91	1.00	0.96	1.00
Germany	0.93	1.00	0.99	1.00
Ghana	0.88	0.99	0.95	1.00
Greece	0.93	1.00	0.96	1.00
Hong Kong	0.94	1.00	0.98	1.00
Hungary	0.92	1.00	0.98	1.00
India	0.86	1.00	0.95	1.00
Indonesia	0.91	1.00	0.95	1.00
Iran	0.86	0.99	0.95	1.00
Italy	0.83	1.00	0.92	1.00
Kazakhstan	0.74	0.98	0.93	1.00
Kenya	0.92	1.00	0.94	1.00
Malaysia	0.93	0.99	0.89	1.00
Mexico	0.91	1.00	0.97	1.00
Nigeria	0.87	1.00	0.87	1.00
Pakistan	0.89	0.99	0.87	1.00
Poland	0.93	1.00	0.98	1.00
Portugal	0.89	0.99	0.98	1.00
Romania	0.94	0.98	0.97	0.92
Russia	0.87	0.99	0.94	1.00
Saudi Arabia	0.82	0.98	0.92	1.00
Slovakia	0.92	1.00	0.95	1.00
South Korea	0.90	1.00	0.97	1.00
Spain	0.90	1.00	0.96	1.00
Switzerland	0.90	1.00	0.98	1.00
Turkey	0.93	1.00	0.97	1.00
United Kingdom	0.91	1.00	0.94	1.00
Uganda	0.89	0.99	0.98	1.00

*Tucker's phi coefficients were analyzed by comparing factor score loadings of each country with the pooled data*.

#### Potential predictors of marital satisfaction

Participants completed a series of standard questions concerning: (1) gender, (2) age, (3) marriage duration in years (4) number of children and number of raised children, (5) religiosity and religious affiliation, (6) subjective economic status (7) education, (8) individual level of collectivistic values, and (9) cultural level of individualism.

Religiosity was measured using a single item (“Are you religious?”), and responses ranged from 1 (*not at all*) to 7 (*extremely religious*). Economic status was measured by asking participants to rate their material situation on a 5-point scale—1 (*much better than average in my country)*, 5 (*much worse than average in my country*).

Perceived level of country Collectivism - Individualism was measured by a scale taken from the GLOBE survey (global study on different variables across 62 countries; House et al., 1999). Because our study concerned family, we used only items regarding familial collectivism (Family Collectivistic Practices; House et al., 1999). This scale was created to test pride in and loyalty to family (and/or organization) and family (and/or organizational) cohesiveness. Sample questions from this scale are: “In this society, parents take pride in the individual accomplishments of their children,” “In this society, aging parents generally live at home with their children.” Participants answered this sentence on a 7-point scale (from 1—*strongly agree* to 7—*strongly disagree*). We recoded the answers so that a higher number indicated higher collectivism. Because the original items were constructed to test Collectivism on the national level (i.e., “In this society, aging parents generally live at home with their children”), we added also their modified version, measuring collectivism on the individual level (i.e., “I think, aging parents should live at home with their children”). The possible answers in this scale were the same as in its original version (House et al., 1999).

### Strengths and limitations

Compared to previously published cross-cultural studies, the present data set has a number of distinctive features: (1) our data set involves thousands (*N* = 7,178) of participants allowing large-scale analyses; (2) we considered five different regions of the world, some of which have only been included in a handful of previous studies (e.g., Onyishi et al., 2012); (3) all participants filled in the same questionnaires and almost all of them followed the same procedures; (4) all participants took part in the study in the same years (2012-2013) to control for any temporal effects; and (5) we measured many variables previously shown to correlate with marital satisfaction. To facilitate the further analyses, we provide basic descriptive statistics of the measured variables (see Table 3).

**Table 3:** Descriptive Statistics (average age, marriage duration, education, number of children, marital satisfaction, and collectivistic values).

**Country**	**Number of participants**		**Age *M* (*SD*)**	**Marriage duration *M* (*SD*)**	**Education *M (SD)***	**Number of children *M* (*SD*)**	**Marital satisfaction MRQ scale *M (SD)***	**Marital Satisfaction KMSS scale *M (SD)***	**Collectivism-Individualism—national level *M (SD)***	**Collectivism-Individualism—individual level *M (SD)***
	**F**	**M**								
Brazil	180	301	36.4 (10.3)	10.5 (9.9)	4.57 (0.75)	1.1 (1.2)	4.64 (0.50)	17.3 (3.8)	11.8 (6.0)	10.5 (6.5)
Bulgaria	39	63	38.6 (9.0)	8.8 (6.6)	4.65 (0.77)	1.1 (0.5)	3.94 (0.61)	17.2 (1.6)	8.4 (1.9)	10.2 (2.3)
Canada	44	25	38.7 (10.4)	11.8 (9.4)	4.64 (0.57)	0.8 (1.0)	4.42 (0.82)	16.7 (5.1)	12.7 (2.5)	16.5 (3.2)
China	72	47	33.2 (6.4)	7.6 (6.7)	4.49 (1.02)	1.0 (0.5)	4.47 (0.61)	17.8 (3.4)	10.4 (4.4)	10.7 (4.2)
Croatia	311	300	44.6 (11.6)	18.0 (11.8)	3.99 (0.98)	1.7 (1.1)	4.40 (0.60)	17.3 (3.8)	12.1 (3.5)	11.8 (3.8)
Estonia	99	51	42.6 (12.2)	17.0 (12.6)	4.45 (0.81)	1.9 (1.1)	4.49 (0.59)	17.5 (3.7)	11.9 (3.8)	11.0 (3.6)
Germany	59	42	47.7 (12.5)	17.7 (15.3)	4.17 (1.03)	1.7 (1.0)	4.56 (0.62)	15.3 (5.8)	14.3 (3.8)	13.7 (3.6)
Ghana	50	53	40.3 (9.5)	12.0 (9.6)	4.24 (1.06)	2.5 (1.5)	4.68 (0.50)	17.7 (4.4)	8.4 (3.2)	8.0 (3.2)
Greece	49	46	38.8 (9.0)	11.6 (9.8)	4.21 (0.80)	1.5 (1.0)	4.48 (0.69)	17.4 (3.8)	11.1 (3.8)	12.6 (4.7)
Hong Kong	43	56	47.0 (10.0)	20.3 (10.5)	3.88 (0.96)	1.5 (1.1)	4.01 (0.91)	15.8 (4.9)	12.0 (2.9)	11.6 (3.2)
Hungary	161	75	37.8 (9.5)	12.6 (9.5)	4.08 (0.93)	1.6 (1.0)	4.40 (0.67)	15.9 (4.8)	19.7 (3.6)	18.8 (3.8)
India	164	135	34.1 (8.0)	7.6 (7.4)	4.94 (0.26)	1.0 (0.8)	4.75 (0.40)	18.5 (3.5)	8.7 (3.6)	8.6 (4.3)
Indonesia	64	24	41.8 (9.6)	16.1 (10.8)	4.51 (0.95)	2.0 (1.1)	4.58 (0.65)	18.0 (4.2)	7.2 (3.4)	6.5 (3.3)
Iran	342	263	38.8 (10.8)	15.3 (11.1)	3.68 (1.14)	2.0 (1.5)	4.09 (0.79)	16.5 (4.8)	9.4 (4.0)	9.1 (4.2)
Italy	193	123	48.6 (10.9)	24.6 (11.5)	4.0 (0.8)	1.7 (0.9)	4.61 (0.43)	18.3 (3.3)	11.2 (3.2)	11.0 (3.7)
Kazakhstan	60	60	37.0 (8.3)	13.0 (7.4)	4.3 (1.0)	1.9 (0.6)	4.74 (0.31)	18.2 (3.2)	9.2 (3.2)	8.0 (3.0)
Kenya	47	47	32.4 (7.3)	7.6 (6.0)	4.40 (1.0)	1.8 (1.2)	4.66 (0.57)	17.1 (3.8)	10.4 (4.9)	10.6 (5.1)
Malaysia	50	49	40.0 (8.9)	13.5 (9.2)	4.5 (0.7)	2.9 (2.0)	4.85 (0.36)	19.4 (2.6)	8.0 (2.6)	6.3 (2.1)
Mexico	85	83	38.8 (11.4)	11.7 (9.8)	4.0 (1.8)	1.6 (1.1)	4.65 (0.61)	16.6 (5.0)	10.0 (3.6)	10.32 (4.1)
Nigeria	293	310	38.9 (9.0)	10.4 (8.8)	4.3 (0.9)	2.5 (1.8)	4.71 (0.48)	18.3 (3.7)	9.2 (4.0)	9.6 (4.4)
Pakistan	71	60	35.9 (10.4)	10.3 (9.6)	4.8 (0.6)	1.8 (1.4)	4.54 (0.56)	17.7 (3.5)	8.4 (3.9)	8.1 (3.9)
Poland	278	166	40.5 (11.6)	16.3 (11.9)	4.4 (0.7)	1.8 (1.2)	4.44 (0.69)	14.8 (4.3)	10.9 (3.7)	10.4 (3.8)
Portugal	180	101	46.1 (11.0)	20.8 (12.2)	3.8 (1.0)	1.6 (0.8)	4.63 (0.49)	17.0 (4.8)	8.7 (3.0)	7.1 (2.5)
Romania	47	6	35.2 (6.8)	8.3 (6.6)	4.9 (0.6)	0.9 (0.8)	4.31 (0.90)	16.4 (5.0)	11.0 (3.7)	15.5 (4.9)
Russia	103	121	38.6 (13.9)	13.8 (13.2)	4.6 (0.9)	1.0 (0.8)	4.48 (0.57)	16.9 (4.1)	11.2 (3.3)	11.0 (4.3)
Saudi Arabia	112	81	36.1 (8.3)	12.3 (8.5)	4.6 (0.8)	2.8 (1.7)	3.91 (0.65)	15.8 (4.5)	6.6 (3.1)	6.6 (3.4)
Slovakia	157	77	42.7 (11.8)	18.3 (11.9)	4.5 (0.6)	1.8 (1.0)	4.26 (0.78)	16.3 (4.6)	10.8 (3.3)	11.2 (3.4)
South Korea	50	50	41.8 (7.7)	15.0 (8.2)	4.4 (0.6)	1.7 (0.8)	4.36 (0.56)	16.7 (3.7)	11.56 (3.6)	10.9 (3.8)
Spain	108	92	47.1 (9.4)	19.4 (10.1)	3.8 (1.1)	1.8 (0.9)	4.54 (0.60)	17.2 (4.0)	11.5 (3.4)	11.1 (3.3)
Switzerland	68	104	49.4 (12.4)	21.7 (13.0)	4.4 (0.6)	2.0 (1.3)	4.54 (0.55)	15.9 (5.4)	15.9 (2.9)	15.9 (3.6)
Turkey	153	239	42.7 (13.6)	16.6 (13.8)	4.1 (1.1)	1.7 (1.2)	4.40 (0.66)	17.7 (10.0)	8.7 (3.3)	9.6 (4.2)
United Kingdom	58	42	45.0 (11.6)	19.4 (13.1)	4.3 (0.7)	1.7 (1.4)	4.61 (0.47)	19.0 (2.8)	12.8 (3.2)	10.8 (3.5)
Uganda	39	62	34.9 (9.9)	8.2 (8.2)	4.1 (1.0)	2.7 (2.1)	4.49 (0.59)	16.2 (4.5)	12.6 (4.1)	11.3 (4.4)
In total	3,827	3,351	40.7 (11.4)	14.8 (11.6)	4.2 (0.9)	1.8 (1.3)	4.47 (0.64)	17.2 (4.2)	10.7 (4.5)	10.5 (4.7)

Despite the numerous strengths, our study has some limitations. Firstly, due to sampling procedures it could have been the case that both partners in the relationship completed the survey. There is no way to be certain about this, but it is unlikely that multiple individuals within relationship jointly participated in the study which might potentially cause issues related to the interdependence of the data. However, even if both partners took part in the study, their answers did not influence each other, because when both a wife and a husband were taking part in the research, they were completing their questionnaires separately. We were highly concerned with our participants' anonymity and sincerity. Secondly, our sample might not be fully representative of the participating countries, as data was collected in particular sites.

### Possible research paths

Based on the presented dataset, scientists can conduct numerous analyses and publish articles concerning various research questions: they can examine cross-cultural differences in marital satisfaction, identifying other country-level predictors of marital satisfaction or use the measures of individualism/collectivism provided in the dataset. These potential country-level predictors (for example shared values in a culture given or demographic data) are likely to be obtainable from other online sources. These may include for example Schwartz's value orientations (Schwartz, 2006) or Hofstede's culture dimensions (Hofstede, 2001). Further, they can examine the indirect replicability of previously conducted studies of correlates of marital satisfaction. Although differences in marital satisfaction have been investigated in a number of cross-cultural and cross-ethnic studies, due to the vast amount of data from this set, the data may also serve as a reference point in further studies regarding marital satisfaction. The dataset can be used for purposes of methodological papers about the validity of existing marriage satisfaction scales (their psychometric properties across different countries).

One previously published study has been based on the presented dataset. Hilpert et al. (2016) found a culturally differentiated association between dyadic coping and marriage satisfaction. They also tested whether gender might moderate the association and found that in some nations the association is higher for men and in other nations it is higher for women.

### Dataset description

The data discussed in this manuscript have been deposited in Figshare repository and is accessible through the following hyperlink: https://figshare.com/s/d2bd33a9605a3a204881 under the name: “*Marital, Sex, Age, Marriage Duration, Religion, Number of Children, Economic Status, Education, and Collectivistic Values: Data from 33 Countries.”* The deposit contains two files: (1) Marital satisfaction_Data, a xlsx file containing the raw data, and (2) Marital satisfaction_Questionnaire, a doc file containing the questionnaire, along with an exhaustive description of the column labels in the dataset.

## Ethics statement

This study was carried out in accordance with the recommendations of Institutional Review Board of the University of Wroclaw with written informed consent from all subjects. All subjects gave written informed consent in accordance with the Declaration of Helsinki. The protocol was approved by the Institutional Review Board.

## Author contributions

All authors listed, have made substantial, direct and intellectual contribution to the work, and approved it for publication. PS, AR, PH, and AS designed the study, PS, AS, AG, TF, KC, AR, PH contributed to the preparation of the manuscript. PS and AS coordinated the project. The rest of the authors collected data.

### Conflict of interest statement

The authors declare that the research was conducted in the absence of any commercial or financial relationships that could be construed as a potential conflict of interest.
