# Corrigendum: Marital Satisfaction, Sex, Age, Marriage Duration, Religion, Number of Children, Economic Status, Education, and Collectivistic Values: Data from 33 Countries

**DOI:** 10.3389/fpsyg.2017.01728

**Published:** 2017-10-05

**Authors:** Piotr Sorokowski, Ashley K. Randall, Agata Groyecka, Tomasz Frackowiak, Katarzyna Cantarero, Peter Hilpert, Khodabakhsh Ahmadi, Ahmad M. Alghraibeh, Richmond Aryeetey, Anna Bertoni, Karim Bettache, Marta Błazejewska, Guy Bodenmann, Tiago S. Bortolini, Carla Bosc, Marina Butovskaya, Felipe N. Castro, Hakan Cetinkaya, Diana Cunha, Daniel David, Oana A. David, Fahd A. Dileym, Alejandra C. Domínguez Espinosa, Silvia Donato, Daria Dronova, Seda Dural, Maryanne Fisher, Aslihan Hamamcıoğlu Akkaya, Takeshi Hamamura, Karolina Hansen, Wallisen T. Hattori, Ivana Hromatko, Evrim Gülbetekin, Raffaella Iafrate, Bawo James, Feng Jiang, Charles O. Kimamo, Firat Koç, Anna Krasnodębska, Amos Laar, Fívia A. Lopes, Rocio Martinez, Norbert Meskó, Natalya Molodovskaya, Khadijeh Moradi Qezeli, Zahrasadat Motahari, Jean C. Natividade, Joseph Ntayi, Oluyinka Ojedokun, Mohd S. B. Omar-Fauzee, Ike E. Onyishi, Barış Özener, Anna Paluszak, Alda Portugal, Anu Realo, Ana P. Relvas, Muhammad Rizwan, Agnieszka L. Sabiniewicz, Svjetlana Salkicević, Ivan Sarmány-Schuller, Eftychia Stamkou, Stanislava Stoyanova, Denisa Šukolová, Nina Sutresna, Meri Tadinac, Andero Teras, Edna L. T. Ponciano, Ritu Tripathi, Nachiketa Tripathi, Mamta Tripathi, Maria E. Yamamoto, Gyesook Yoo, Agnieszka Sorokowska

**Affiliations:** ^1^Institute of Psychology, University of Wroclaw, Wroclaw, Poland; ^2^Counseling and Counseling Psychology, Arizona State University, Tempe, AZ, United States; ^3^Faculty in Sopot, SWPS University of Social Sciences and Humanities, Sopot, Poland; ^4^Department of Psychiatry and Behavioral Science, University of Washington, Seattle, DC, United States; ^5^Behavioral Sciences Research Center, Baqiyatallah University of Medical Sciences, Tehran, Iran; ^6^Department of Psychology, King Saud University, Riyadh, Saudi Arabia; ^7^School of Public Health, University of Ghana, Legon, Ghana; ^8^Department of Psychology, Catholic University of Milan, Milan, Italy; ^9^Department of Psychology, The Chinese University of Hong Kong, Hong Kong, Hong Kong; ^10^Department of Psychology, University of Zurich, Zurich, Switzerland; ^11^Graduate Program in Morphological Sciences, Federal University of Rio de Janeiro, Rio de Janeiro, Brazil; ^12^Cognitive and Behavioral Neuroscience Unit, D'Or Institute for Research and Education, Rio de Janeiro, Brazil; ^13^Institute of Ethnology and Anthropology, Russian Academy of Sciences, Moscow, Russia; ^14^Russian State University for the Humanities, Moscow, Russia; ^15^Laboratory of Evolution of Human Behavior, Federal University of Rio Grande do Norte, Natal, Brazil; ^16^Department of Psychology, Faculty of Languages History and Geography, Ankara University, Ankara, Turkey; ^17^Faculty of Psychology and Education Sciences, University of Coimbra, Coimbra, Portugal; ^18^International Institute for the Advanced Studies of Psychotherapy and Applied Mental Health, Babes-Bolyai University, Cluj-Napoca, Romania; ^19^Department of Clinical Psychology and Psychotherapy, Babes-Bolyai University, Cluj-Napoca, Romania; ^20^Department of Psychology, Universidad Iberoamericana, Mexico City, Mexico; ^21^Faculty of Arts and Sciences, Izmir University of Economics, Izmir, Turkey; ^22^Department of Psychology, Saint Mary's University, Halifax, Canada; ^23^Department of Anthropology, Cumhuriyet University, Sivas, Turkey; ^24^Faculty of Psychology, University of Warsaw, Warsaw, Poland; ^25^Faculty of Medicine, Federal University of Uberlândia, Uberlândia, Brazil; ^26^Department of Psychology, University of Zagreb, Zagreb, Croatia; ^27^Department of Psychology, Akdeniz University, Antalya, Turkey; ^28^Department of Clinical Services, Federal Neuro-Psychiatric Hospital, Benin-City, Nigeria; ^29^Central University of Finance and Economics, Beijing, China; ^30^Department of Psychology, University of Nairobi, Nairobi, Kenya; ^31^Institute of Pedagogical Sciences, University of Opole, Opole, Poland; ^32^Department of Social Psychology, University of Granada, Granada, Spain; ^33^Institute of Psychology, University of Pécs, Pécs, Hungary; ^34^Department of Agricultural Extension and Education, Razi University, Kermanshah, Iran; ^35^Institute of Psychology, University of Science and Culture, Tehran, Iran; ^36^Institute of Psychology, Pontifical Catholic University of Rio de Janeiro, Rio de Janeiro, Brazil; ^37^Faculty of Computing and Management Science, Makerere University Business School, Kampala, Uganda; ^38^Department of Pure & Applied Psychology, Adekunle Ajasin University, Akungba-Akoko, Nigeria; ^39^School of Education and Modern Languages, Universiti Utara Malaysia, Sintok, Malaysia; ^40^Department of Psychology, University of Nigeria, Nsukka, Nigeria; ^41^Department of Anthropology, Istanbul University, Istanbul, Turkey; ^42^Department of Psychology, University of Warwick, Coventry, United Kingdom; ^43^Department of Psychology, University of Tartu, Tartu, Estonia; ^44^University of Karachi, Institute of Clinical Psychology, Karachi, Pakistan; ^45^Center of Social and Psychological Sciences, Institute of Experimental Psychology SAS, Bratislava, Slovakia; ^46^Department of Social Psychology, University of Amsterdam, Amsterdam, Netherlands; ^47^Department of Psychology, South-West University “Neofit Rilski”, Blagoevgrad, Bulgaria; ^48^Educational Research Center, Matej Bel University, Banská Bystrica, Slovakia; ^49^Coaching Department, Universitas Pendidikan Indonesia, Bandung, Indonesia; ^50^Mõttemaru OÜ, Tartu, Estonia; ^51^Institute of Psychology, University of the State of Rio de Janeiro, Rio de Janeiro, Brazil; ^52^Indian Institute of Management Bangalore, Organizational Behaviour and Human Resource Management, Bangalore, India; ^53^Indian Institute of Technology Guwahati, Guwahati, India; ^54^Department of Child and Family Studies, Kyung Hee University, Seoul, South Korea; ^55^Department of Psychotherapy and Psychosomatic Medicine, Technische Universität Dresden, Dresden, Germany

**Keywords:** marital satisfaction, cross-cultural research, relationships, Religion and Psychology, family studies

In the published article, there was an error in affiliation 38. Instead of “Faculty of Social and Management Sciences, Adekunle Ajasin University, Akungba-Akoko, Nigeria,” it should be “Department of Pure and Applied Psychology, Adekunle Ajasin University, Akungba-Akoko, Nigeria.” Also, the affiliation of Fívia A. Lopes is “Laboratory of Evolution of Human Behavior, Federal University of Rio Grande do Norte, Natal, Brazil” instead of “Department of Psychology, Faculty of Languages History and Geography, Ankara University, Ankara, Turkey.”

Fahd A. Dileym was not included as an author in the published article. The authors apologize for these errors and state that this does not change the scientific conclusions of the article in any way.

## Conflict of interest statement

The authors declare that the research was conducted in the absence of any commercial or financial relationships that could be construed as a potential conflict of interest.

